# Human Alpha-1-Antitrypsin (hAAT) therapy reduces renal dysfunction and acute tubular necrosis in a murine model of bilateral kidney ischemia-reperfusion injury

**DOI:** 10.1371/journal.pone.0168981

**Published:** 2017-02-24

**Authors:** Nuria Maicas, Johan van der Vlag, Janin Bublitz, Sandrine Florquin, Marinka Bakker-van Bebber, Charles A. Dinarello, Vivienne Verweij, Roos Masereeuw, Leo A. Joosten, Luuk B. Hilbrands

**Affiliations:** 1 Department of Nephrology, Radboud University Medical Center, Nijmegen, the Netherlands; 2 Department of Pathology, Radboud University Medical Center, Nijmegen, the Netherlands; 3 Department of Internal Medicine, Radboud University Medical Center, Nijmegen, the Netherlands; 4 Department of Medicine, University of Colorado Health Sciences Center Denver, Colorado, United States of America; 5 Department of Pharmacology and Toxicology, Radboud University Medical Center, Radboud Institute for Molecular Life Sciences, Nijmegen, the Netherlands; 6 Division of Pharmacology, Utrecht Institute for Pharmaceutical Sciences, Utrecht, the Netherlands; Hopital Tenon, FRANCE

## Abstract

Several lines of evidence have demonstrated the anti-inflammatory and cytoprotective effects of alpha-1-antitrypsin (AAT), the major serum serine protease inhibitor. The aim of the present study was to investigate the effects of human AAT (hAAT) monotherapy during the early and recovery phase of ischemia-induced acute kidney injury. Mild renal ischemia-reperfusion (I/R) injury was induced in male C57Bl/6 mice by bilateral clamping of the renal artery and vein for 20 min. hAAT (80 mg/kg, Prolastin^®^) was administered daily intraperitoneally (i.p.) from day -1 until day 7 after surgery. Control animals received the same amount of human serum albumin (hAlb). Plasma, urine and kidneys were collected at 2h, 1, 2, 3, 8 and 15 days after reperfusion for histological and biochemical analysis. hAAT partially preserved renal function and tubular integrity after induction of bilateral kidney I/R injury, which was accompanied with reduced renal influx of macrophages and a significant decrease of neutrophil gelatinase-associated lipocalin (NGAL) protein levels in urine and plasma. During the recovery phase, hAAT significantly decreased kidney injury molecule-1 (KIM-1) protein levels in urine but showed no significant effect on renal fibrosis. Although the observed effect size of hAAT administration was limited and therefore the clinical relevance of our findings should be evaluated carefully, these data support the potential of this natural protein to ameliorate ischemic and inflammatory conditions.

## Introduction

Renal ischemia-reperfusion (I/R) injury is a frequent cause of acute kidney injury (AKI) in a variety of clinical conditions such as cardiac or aortic surgery and kidney transplantation, and is associated with significant morbidity and mortality. Ischemic injury of the kidney causes the release of damage-associated molecular patterns (DAMPs) by impaired endothelial and tubular epithelial cells [1]. These DAMPs are rapidly sensed by pattern recognition receptors, which together with the formation of reactive oxygen species after reperfusion lead to an inflammatory response. Therefore, the focus of current research is to control innate immune pathways in order to reduce I/R damage.

Alpha-1-antitrypsin (AAT) is the most abundant serum serine protease inhibitor, primarily produced by the liver. Its main physiological role is to inhibit the activity of different endogenous serine proteases, such as neutrophil-derived elastase and proteinase-3 [2–4]. These proteolytic enzymes contribute to the inflammatory response by activating pro-cytokines and through the formation of DAMPs. Due to its anti-protease activity, AAT can inhibit these pathways, consequently exerting anti-inflammatory and tissue-protective effects. During acute-phase responses, such as injury or infection, circulating AAT levels can increase approximately four-fold. In addition, AAT levels are up-regulated during hypoxia [5] and are known to be increased in plasma from patients with acute myocardial infarction [6, 7] and in urine of patients with AKI [8], probably as an endogenous protective response against ischemic injury. Accordingly, human AAT (hAAT) given to mice during acute myocardial I/R injury limited the infarct size and protected from adverse cardiac remodeling [9]. Moreover, there is a growing body of evidence supporting the anti-inflammatory and cytoprotective effects of this acute-phase reactant in a wide range of *in vitro* and *in vivo* experimental models [10]. The cellular targets of AAT mostly include cells of the innate immune system, such as neutrophils and macrophages, as well as B lymphocytes and dendritic cells which are involved in the adaptive immune response. However, its mechanism of action is not completely understood, and some studies suggest that the protective effects of AAT are independent of its serine protease inhibiting activity [11].

Given the pivotal role of the early inflammatory response in the pathogenesis of ischemic injury, we sought to investigate the effects of hAAT monotherapy on both AKI and the kidney repair process after ischemic insult. To address these issues we performed a mouse model of bilateral kidney I/R injury.

## Materials and methods

### Animals

All animal procedures were approved by the Animal Ethics Committee of the Radboud university (Nijmegen, the Netherlands; RU-DEC 2011–049 / 2013–198). Handling of animals was performed according to the guidelines of the Dutch Council for Animal Care and the European Communities Council Directive (86/609/EEC). Male C57Bl/6N mice (Charles River, Sulzfeld, Germany) were housed at the Central Animal Facility of the Radboud University under specific pathogen-free conditions with *ad libitum* food and water.

### Experimental bilateral kidney I/R model

All surgical procedures were performed on 8/9-week-old mice (22–28 g) using standard aseptic surgical techniques, with all efforts to minimize suffering. Carprofen [5 mg/kg body weight (b.w.)] was selected as a non-steroidal analgesic in all experimental groups and administered subcutaneously (s.c.) 30 min before the surgery, 24h and 48h after surgery. Anesthesia was induced with 5% isoflurane in O_2_/N_2_O and subsequently kept at 2.5–3% during the operation. Mice were laparotomized and body temperature was maintained at 36.5–37°C. The renal vein and artery of both kidneys were freed from surrounding white adipose tissue and clamped with microvascular clamps (B-1V from S&T, Neuhausen, Switzerland) for 20 min. Absence of renal blood flow during clamping and subsequent renal reperfusion after releasing the clamp, was visually monitored by respectively the discoloring and re-coloring of the kidney. Animals that did not display a homogeneous and marked kidney color change or with high temperature (≥ 38°C) during the surgical procedure were excluded from the study. In a pilot study to determine the appropriate ischemic time for this model within our experimental conditions, a sham-operation group (*n* = 3 animals) was included. Same surgical procedure, without clamping of the renal vessels, was performed on these animals. Sham-operated mice overcame the surgery without neither signs of sickness nor renal changes when compared to naïve animals: levels of plasma creatinine (<12 μmol/L *vs* naïve animals <12 μmol/L), urine KIM-1 levels (429.0±190.9 pg/mL *vs* naïve animals 406.1±136.7 pg/mL; *P*>0.05) and plasma NGAL levels (241.3±77.9 ng/mL *vs* naïve animals 161.3±41.1 ng/mL; *P*>0.05).

### Study design and treatment

Mice were randomly assigned to the experimental groups (*n* = 6–8 animals per control groups and *n* = 6–8 animals per hAAT groups) and placed in metabolic cages around 1 week before surgery (day 7 pre-op), immediately after the surgery, and at day 1, 2, 7, or 14 after reperfusion (post-op) to collect urine. Blood samples were obtained and mice were sacrificed by cervical dislocation at 2h and 1, 2, 3, 8, and 15 days after surgery.

Clinical grade human AAT (hAAT, Prolastin^®^, Bayer Corporation) was dissolved in sterile water and administered intraperitoneally (i.p.) at a dose of 80 mg/kg (2 mg/mouse/day; injection volume of 200 μL) starting at day -1 (24h before the surgery), day 0 (30 min before the surgery) and then daily for a maximum of 7 days. Control animals received the same amount of human serum albumin (hAlb; injection volume of 200 μL) (Sigma-Aldrich) as control for human protein administration. Weight and well-being of the mice were monitored daily.

### Tissue and blood handling

Blood samples were collected in heparin tubes and centrifuged at 1200 *xg* for 10 min at 10°C to obtain plasma. Protease inhibitors were added to urine samples after centrifugation at 3000 *xg* for 15 min at 4°C. Plasma and urine samples were stored at -20°C and -80°C, respectively. Kidneys were harvested and fixed in 4% paraformaldehyde in order to perform the histological analysis or snap frozen in liquid nitrogen to perform the immunohistochemical analysis and RNA isolation.

### Renal function assessment

The renal function was determined by measuring creatinine and urea levels in plasma samples using routine standard clinical chemical methods by our hospital diagnostic facility.

### Histological analysis

Fixed renal tissue was dehydrated and subsequently embedded in paraffin. Kidneys were cut at different latitudes into 4-μm sections, mounted on 3-aminopropyltriethoxysilase (APES)-coated slides and dried at 37°C for at least 24h. Sections were stained with periodic acid-Schiff (PAS). All the histopathological scores were made in 3 different areas: outer cortex (OC), cortico-medullary junction (CMJ) and inner medulla (IM). The percentage of damaged tubules in these 3 areas was estimated as described previously [12] and according to the following criteria: cast formation, debris deposition in the tubular lumen and loss of the brush border from the proximal tubules in 10 randomly chosen, non-overlappig fields (*x400* magnification). Lesions were graded on a scale from 0 to 5: 0 = normal, no proximal tubular damage; 1 = mild, involvement of less than 10% of the area; 2 = moderate, involvement of 10–25% of the area; 3 = severe, involvement of 25–50% of the area; 4 = very severe, involvement of 50–75% of the area; 5 = extensive damage, involvement of more than 75% of the area. The score was performed on blinded sections by two observers.

### Kidney fibrosis

Tissue sections (4 μm) were deparaffinized, rehydrated and stained with 0.2% Picro-Sirius Red for 60 min for assessment of fibrotic tissue formation. Collagen fibers were coloured in bright red on a pale yellow background. Quantification of collagen-stained area (μm^2^) was performed in non-overlapping fields (*x200* magnification) throughout 2 different regions of the kidney (outer cortex and cortico-medullary junction) using the image software KS 400, and subsequently averaged. Analysis was performed on blinded sections by an observer.

### Measurement of AKI markers in urine and plasma

Kidney injury molecule-1/T-cell Ig mucin protein-1 (KIM-1/TIM-1) and neutrophil gelatinase-associated lipocalin (NGAL) protein levels were measured in urine and plasma using high sensitivity enzyme-linked immunosorbent assay (ELISA) kits (from R&D Systems Inc., Minneapolis, MN, USA).

### Measurement of plasma hAAT levels and mouse anti-hAAT antibodies

Determination of plasma hAAT levels was performed using an ELISA kit (GenWay Biotech, Inc., San Diego, CA, USA). Detection of mouse anti-hAAT antibody levels in plasma was performed by ELISA as previously described [13].

### Determination of inflammatory cells by Immunofluorescense (IF)

Frozen kidney sections (2 μm) were analyzed by IF as described previously [14]. Double stainings with a monoclonal antibody against the core protein of agrin (hamster anti-mouse; MI91) [15] (dilution 1:800) and against granulocytes (rat anti-mouse; Gr-1, Ly6G/LY-6C-FITC labeled) (BioLegend, San Diego, CA, USA) (dilution 1:100) or macrophages (rat anti-mouse; CD68) (AbD Serotec, Bio-Rad laboratories, Inc., Hercules, CA, USA) (dilution 1:1600) were performed. CD68 and MI91 were stained with goat anti-rat Alexa Fluor^®^488-conjugated secondary antibody (Invitrogen, Paisley, UK) (dilution 1:200) and goat anti-hamster Cy^™^3-conjugated secondary antibody (Jackson ImmunoResearch Laboratories, PA, USA) (dilution 1:600), respectively. Granulocytes were counted in the kidney cortex sections in non-overlapping fields (*x400* magnification) and macrophage staining was scored semi-quantitatively on a scale from 0 to 5 based on the extent of CD68 immunofluorescence staining by two observers on blinded sections.

### RNA isolation from kidney tissue and real-time quantitative RT-qPCR

RNA was isolated from kidney tissue using the RNeasy Mini kit (Qiagen) and reverse transcribed (Transcription Kit; Roche Diagnostics, Mannheim, Germany). Interleukin-1β (IL-1β), macrophage galactose-type C-type lectin-1 (MGL-1), macrophage inflammatory protein-1α (MIP-1α), monocyte chemoattractant protein-1 (MCP-1), interferon regulatory factor 5 (IRF5), chemokine (C-X-C motif) receptor 2 (CXCR2), mouse keratinocyte-derived cytokine (KC), transforming growth factor-β (TGF-β), collagen-1α (col-1α), collagen-4 (col-4) and matrix metalloproteinase-9 (MMP-9) gene expression was determined by real-time quantitative PCR using SYBR Green SuperMix (Roche Diagnostics) on a MyiQ real-time PCR detection system (Bio-Rad Laboratories), as described previously [16]. Primer sequences for the housekeeping gene glyceraldehyde-3-phosphate dehydrogenase (GAPDH) and the genes of interest can be found in S1 Table. For each sample, differences in threshold cycle (ΔCt) values were calculated by correcting the Ct of the gene of interest to the Ct of the housekeeping gene GAPDH. Results were expressed as Rq (fold change) = 2^-ΔCt (hAAT group)^ / mean 2^-ΔCt (control group)^.

### Statistical analysis

Values are represented as box and whisker plots with median, 25^th^ and 75^th^ percentiles, and minimum and maximum values, or as mean±SEM. Data were analyzed using GraphPad Prism^®^ (version 5.03 for Windows; Graphpad Software Inc., San Diego, CA). Spearman´s correlation coefficient was used to quantify the relationship between two variables. Differences between experimental groups and changes of parameters in time were tested using the two-tailed Mann-Whitney U test when comparing two treatment groups at one time-point or two-way analysis of variance (ANOVA) of data after logarithmic normalization (factors time and treatment) followed by *Bonferroni* multiple comparison post-test, as appropriate (see Figure legends). *P* values less than 0.05 were considered significant.

## Results

### hAAT protects against I/R-induced renal dysfunction

**Fig 1:**
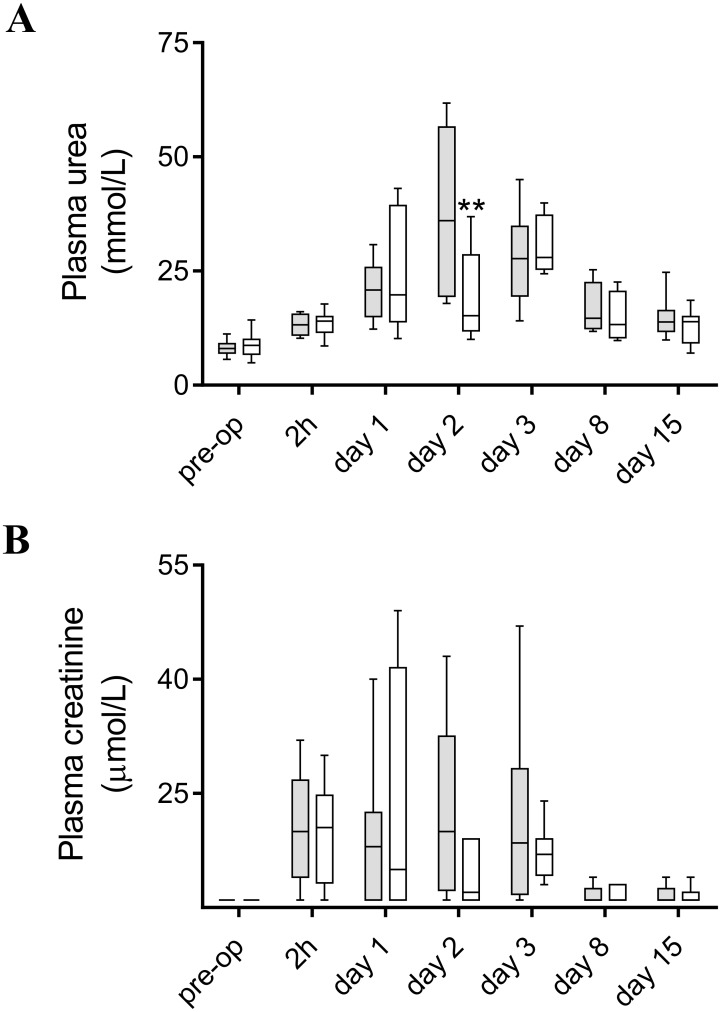
hAAT (80 mg/kg/day; i.p.) treatment improves renal function after I/R Injury (IRI). **A.** Plasma urea levels at day 7 pre-op and at different time-points after IRI. **B.** Plasma creatinine levels at day 7 pre-op and at different time-points after IRI. Grey boxes represent the control group treated with hAlb. White boxes represent the group treated with hAAT. Two-way analysis of variance (ANOVA) followed by *Bonferroni* post-test. **P<0.01 (*n* = 6–8 animals per group).

I/R injury significantly impaired kidney function, as reflected by a marked increase in plasma creatinine and urea levels. The observed increase of these parameters peaked at days 2 and 3 after reperfusion, followed by a return to basal levels with almost complete recovery at day 15 post-op. Treatment with hAAT ameliorated acute renal dysfunction, partly protecting against renal failure as indicated by significantly lower levels of plasma urea at day 2 after reperfusion (Fig 1A) and a tendency to lower plasma creatinine levels compared to the control group (Fig 1B).

### hAAT decreases I/R-induced tubular damage

**Fig 2:**
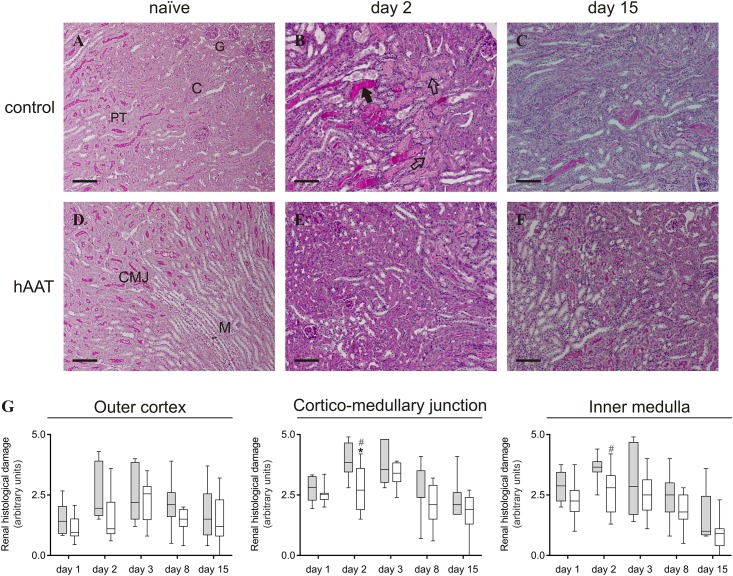
hAAT (80 mg/kg/day; i.p.) treatment decreases acute tubular necrosis after I/R Injury (IRI). **A-F.** PAS-stained frontal sections of mouse kidney: **A, D** PAS-stained frontal sections of naïve mouse kidney without any histological damage and with the intact brush border in the proximal tubules. **B, C** representative images of ischemic kidney of control mouse at different time-points after IRI. **E, F** representative images of ischemic kidney of mouse treated with hAAT at different time-points after IRI. C = cortex; CMJ = cortico-medullary junction; M = medullar region; PT = proximal tubules; G = glomeruli. The solid arrow indicates areas of cast formation. Open arrows indicate debris deposition in the tubular lumen. Scale bar = 100 μm (original magnification *x100*). **G.** Kidney histological damage score. The percentage of damaged tubules in 3 different areas of the kidney was estimated using a 5-point scale. Grey boxes represent the control group treated with hAlb. White boxes represent the group treated with hAAT. Two-way analysis of variance (ANOVA) followed by *Bonferroni* post-test: *P<0.05. Mann-Whitney U test (two-tailed) to analyze the effect of treatment at each time-point. ^#^*P*<0.05 (*n* = 6–8 animals per group).

Two days after inducing I/R injury, kidneys showed widespread tubular damage over the cortex, cortico-medullary junction and medullary region, reflected by a significant increase in the renal damage score (Fig 2G) when compared to naïve animals (Fig 2, panels A and D). These damaged and necrotic tubules showed either cast formation, debris deposition in the tubular lumen, or shedding of the brush border from the proximal tubules (Fig 2B). One week after the ischemic insult, tubules started to recover as can be concluded from the repaired brush border membranes that were observed during the late stages of kidney I/R damage (Fig 2C). Kidneys from hAAT-treated mice displayed less extensive tubular necrosis compared to the hAlb-treated control animals (Fig 2, panels E and F), with a significantly reduced histological damage score in the cortico-medullary junction and inner medulla at day 2 after kidney I/R injury (Fig 2G).

### hAAT ameliorates Acute Kidney Injury (AKI)

**Fig 3:**
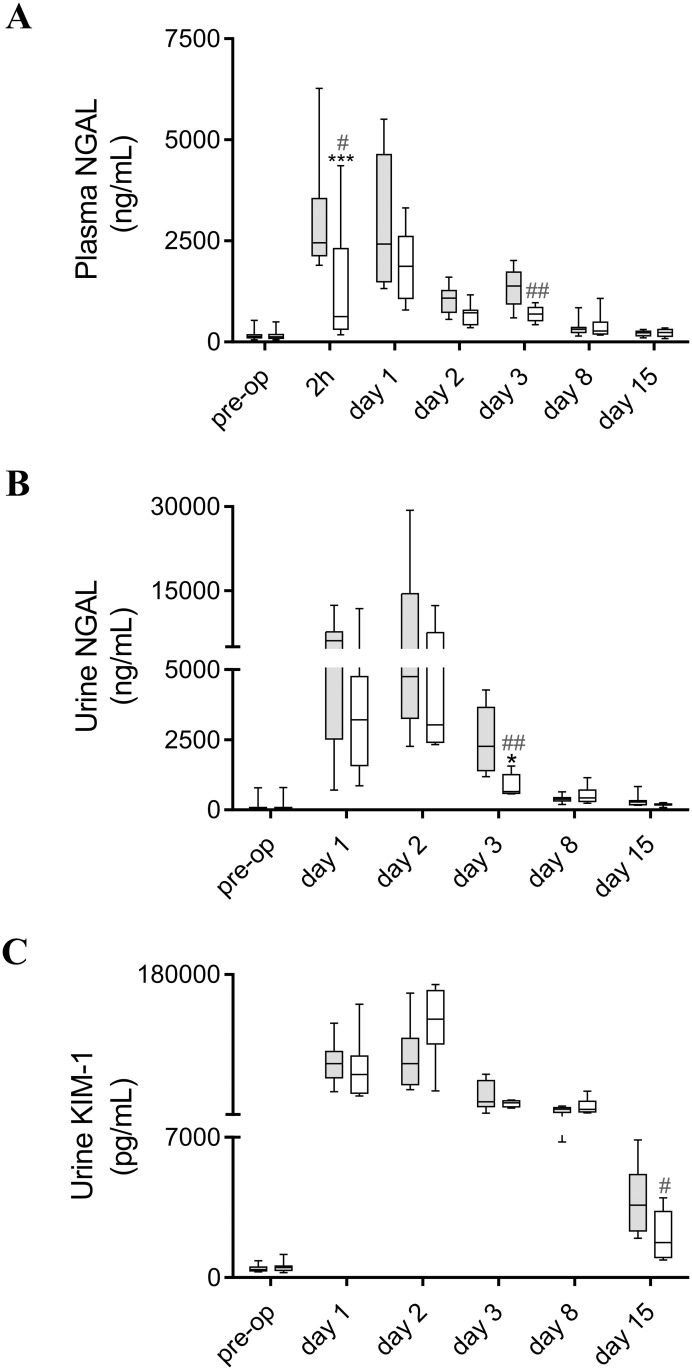
hAAT (80 mg/kg/day; i.p.) treatment ameliorates AKI after I/R Injury (IRI). **A.** Plasma NGAL levels at day 7 pre-op and at different time-points after IRI. **B.** Urine NGAL levels at day 7 pre-op and at different time-points after IRI. **C.** Urine KIM-1 levels at day 7 pre-op and at different time-points after IRI. Grey boxes represent the control group treated with hAlb. White boxes represent the group treated with hAAT. Two-way analysis of variance (ANOVA) followed by *Bonferroni* post-test: *P<0.05, ***P<0.001. Mann-Whitney U test (two-tailed) to analyze the effect of treatment at each time-point. ^#^*P*<0.05, ^##^*P*<0.01 (*n* = 6–8 animals per group).

To assess AKI, we measured the concentration of the acute renal injury markers KIM-1 and NGAL in urine and plasma. When compared to the basal levels at day 7 pre-op, 20-min renal ischemia significantly increased the protein levels of both AKI markers KIM-1 and NGAL in urine and plasma (Fig 3). Maximal expression of these proteins was observed during the early phase of the renal I/R injury.

Treatment with hAAT ameliorated AKI, as indicated by a smaller rise in NGAL protein levels in urine and plasma in the early phase of the kidney I/R damage (Fig 3, panels A and B). In addition, hAAT therapy significantly reduced the urine protein levels of KIM-1 at day 15, during the recovery phase of the renal ischemic damage (Fig 3C).

### Effects of hAAT on granulocyte and macrophage infiltration in post-ischemic kidneys

**Fig 4:**
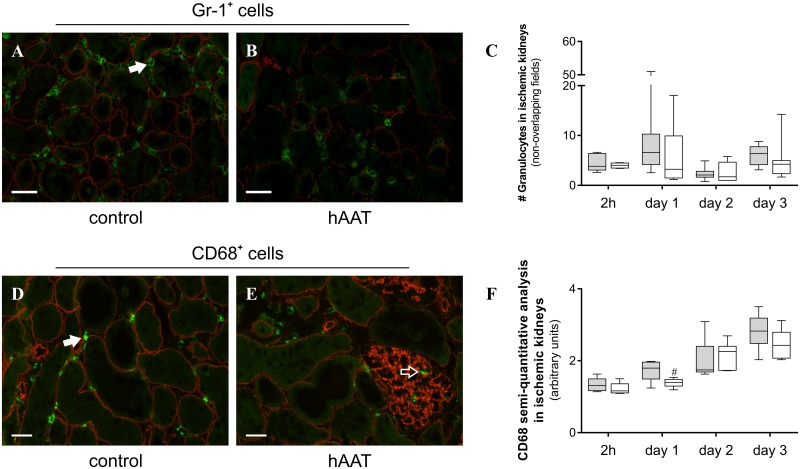
Effect of hAAT (80 mg/kg/day; i.p.) treatment on renal leukocyte influx at different time-points after I/R Injury (IRI). **A, B.** Immunofluorescence analysis of granulocytes in ischemic kidneys. Kidney sections were stained with a specific anti-Gr-1 antibody. **A** representative image of ischemic control mouse kidney at day 1 after IRI. **B** representative image of ischemic mouse kidney treated with hAAT at day 1 after IRI. Scale bar = 50 μm (original magnification *x200*). **D, E.** Immunofluorescence analysis of macrophages in ischemic kidneys. Kidney sections were stained with a specific anti-CD68 antibody. **D** representative image of ischemic kidney of a control mouse at day 1 after IRI. **E** representative image of ischemic kidney of a mouse treated with hAAT at day 1 after IRI. Scale bar = 20 μm (original magnification *x400*). Solid arrows indicate intertubular cell infiltration. The open arrow indicates cellular influx in the glomeruli. **C.** Gr-1^+^ cells were counted in non-overlapping fields and subsequently averaged. **F.** Macrophage staining was scored semi-quantitatively on a scale from 0 to 5 based on the extent of CD68 immunofluorescence staining. Grey boxes represent the control group treated with hAlb. White boxes represent the group treated with hAAT (dark green = autofluorescence of the tubuli; red = agrin; bright green = immune cells). Mann-Whitney U test (two-tailed) to analyze the effect of treatment at each time-point. ^#^*P*<0.05 (*n* = 6–8 animals per group).

In order to evaluate the effect of hAAT treatment on leukocyte influx, we evaluated the Gr-1^+^ and CD68^+^ cell staining in post-ischemic kidneys during the early stage of the kidney I/R damage. As depicted in Fig 4, mice subjected to 20-min renal ischemia displayed a significant increase in granulocyte and macrophage infiltration, which peaked at day 1 and 3 after reperfusion, respectively (Fig 4, panels C and F). Immune cell influx was predominantly observed in the intertubular space with only few positive cells in the glomeruli (Fig 4, panels A, B, D and E). At day 1 post-op, there was a significant decrease in renal macrophage influx in hAAT-treated mice in comparison with control animals.

### Effects of hAAT on gene expression in post-ischemic kidneys during the early phase of AKI

The acute inflammatory response triggered by I/R entails the induction of pro-inflammatory chemokines and cytokines together with the concomitant upregulation of CXC receptors. Renal gene expression of the pro-inflammatory cytokine IL-1β, chemokines (MCP-1, MIP-1α and KC), MGL-1, IRF5 and CXCR2 was evaluated at 2h and 1 day post-op. No significant differences were observed between experimental groups (S1 Fig).

### Effect of hAAT on the recovery phase of acute tubular necrosis

Renal function was partly recovered at day 8 after 20-min ischemic insult, as can be concluded from the plasma creatinine and urea levels (S2 Fig). Histological examination of kidney tissues mirrored these findings, with partially restored brush border membranes in proximal tubules at days 8 and 15 post-op (Fig 2C). However, we also observed interstitial fibrosis on collagen staining at days 8 and 15 after reperfusion (S2 Fig, panels A-E), accompanied by an increase (relative to naïve animals) in mRNA levels of col-1α, col-4, MMP-9 and TGF-β. Notably, no significant differences neither in the extent of renal fibrotic tissue (S2A Fig) nor in the expression of these pro-fibrotic genes (S2F Fig) were observed between hAAT-treated animals and hAlb-treated control mice.

### Time-course of circulating levels of hAAT and mouse anti-hAAT antibodies

**Fig 5:**
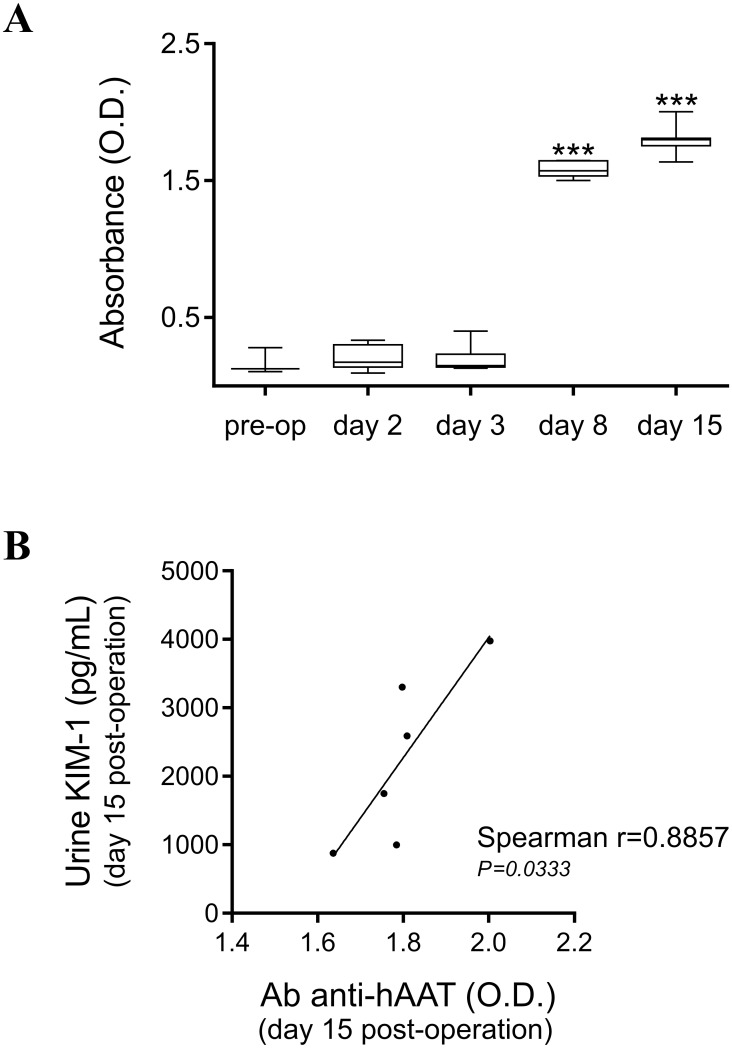
Mouse anti-hAAT antibody formation and its correlation with urine levels of KIM-1 during the recovery phase of renal I/R Injury (IRI). **A.** Mouse anti-hAAT antibody levels in plasma were measured before surgery (pre-op) and at different time-points after IRI. One-way analysis of variance (ANOVA) followed by *Dunnett’s* post-test. ***P<0.001 (*n* = 4–8 animals). **B.** Correlation between protein levels of KIM-1 in urine and mouse anti-hAAT antibodies in serum at day 15 post-op. Spearman´s correlation. *P<0.05 (*n* = 6 animals).

Plasma protein levels of hAAT remained high for at least 8 days after daily i.p. administration of hAAT (532.2±36.81 μg/mL). After discontinuing therapy for 1 week, lower levels of hAAT were detected in the circulation at day 15 post-op (0.161±0.049 μg/mL). Moreover, mouse anti-hAAT antibodies were already detected at day 8 after surgery (Fig 5A).

## Discussion

The current study shows that *in vivo* administration of clinical grade hAAT improves renal function, decreases acute tubular necrosis and ameliorates AKI following experimental kidney I/R damage. Acute renal failure is a severe and potentially life-threatening clinical condition that is often the result of an extended period of renal ischemia, followed by reperfusion. In the kidney transplantation setting, the severity of AKI due to ischemic damage can impact the outcome of the graft [17]. At present, only supportive treatment is available for AKI [18–20], with no therapy available to prevent ischemic damage or to enhance its recovery. Therefore, the protective effects of hAAT observed in our murine model of bilateral renal I/R injury are quite encouraging since it could reduce the incidence of delayed graft function in kidney-transplanted patients and improve the management of acute renal failure in a variety of non-transplant conditions.

In our study, we assessed the effects of daily hAAT administration at different time-points after renal I/R injury. Although endogenous AAT levels may gradually rise as part of an acute phase response after AKI damage [8], early administration of AAT appears to be critical for optimal anti-inflammatory effects [13]. hAAT is cleared at twice the speed in mice compared to humans and therefore frequent injections were required in our experimental model. Daily hAAT administration starting 1 day before surgery until day 7 post-op resulted in sustained plasma levels of the protein. The dosage used in our study was based on earlier mouse studies, where hAAT plasma levels of 0.35–0.45 mg/mL reduced inflammation and allograft rejection in beta cell transplantation [21, 22]. Another *in vivo* study found that i.p. injection of 0.3–1.0 mg AAT protected mice from TNFα-mediated lethal response [23]. Similarly, monotherapy with 2 mg of i.p. AAT prolonged islet allograft survival [13], induced immune tolerance [24] and protected against acute myocardial I/R injury in mice [9].

The pathogenesis of kidney I/R injury is characterized by a complex interaction of renal hemodynamics, tubular damage, and consequent inflammatory responses. In the present work, we observed positive effects of hAAT on kidney morphology, macrophage infiltration and functional alterations in plasma urea levels. Next to these morphological and functional measurements, we also observed reduced levels of the kidney injury markers KIM-1 and NGAL in our experimental model. These proteins are markedly up-regulated in proximal tubule epithelial cells after ischemic damage, and can be detected in the urine of animals and patients with AKI. Furthermore, these proteins were found to be valuable noninvasive biomarkers of renal injury [25–28]. In addition, NGAL has also been investigated in serum for the early diagnosis of acute renal injury [26] and in the transplantation setting strong associations have been found between NGAL levels and biopsy-proven acute rejection [29] or acute kidney allograft dysfunction after living-donor kidney transplantation [30]. We found that KIM-1 was present at low levels in urine of healthy mice, but increased dramatically in urine of post-ischemic animals, reaching a maximum within 24-48h post-op. In agreement with previous studies reporting maximal expression of NGAL at an earlier time-point [26, 27], this protein was up-regulated more than 100-fold in urine and more than 10-fold in plasma, peaking at 2-24h after reperfusion. There was a strong reduction in the early rise in NGAL levels in plasma (*P*<0.001). Levels of urine NGAL and KIM-1 also significantly decreased in hAAT-treated mice during the acute and recovery phase of the I/R injury, respectively. Although NGAL and KIM-1 are both early injury markers, NGAL is longer detectable and a better and more sensitive candidate identifying injuries of the renal tubular system over a wide range of clinical conditions [31]. In mice, these early AKI biomarkers are preferred over creatinine which is not considered the most reliable measure of renal damage in this rodent species. The influence of tubular creatinine excretion on creatinine clearance is known to be larger in mice than in humans, questioning the accuracy of creatinine for determining renal function in these animals [32]. Moreover, although in clinical practice diagnosis of acute renal failure is currently performed by creatinine measurement, it is not a reliable indicator in the setting of acute changes in kidney function [33]. For instance, creatinine levels are likely not to vary until around 50% of kidney function has already been lost and this protein does not give an accurate picture of kidney function until an unfluctuating stage is achieved, which could take approximately various days. Furthermore, creatinine levels can be misleading since it depends on several factors including, but not limited to, muscle mass and metabolism, overall body weight, nutrition and the hydration status of the individual.

Renal tubular epithelial cells that are present in the oxygen-sensitive region of the outer medullar region are highly susceptible to ischemic injury [34]. In the histological analysis of 20-min ischemic kidney, the appearance of casts and debris deposition in the tubular lumen confirmed acute tubular necrosis within 1 day after I/R injury. hAAT treatment ameliorated these renal pathological manifestations. This decrease in tubular damage is most likely the result of less infiltration of innate effector cells and direct inhibition by hAAT of proteolytic enzymes that contribute to the development of injury.

After restoration of the blood flow, ischemic lesions become worse by the reintroduction of oxygen and leukocytes. Neutrophils adhesion to activated endothelium and accumulation in the ischemic kidney has been observed in both animal models and in biopsies from patients with AKI [35, 36]. Neutrophil recruitment takes place as early as 30 minutes after reperfusion, and is normally mediated by adhesion molecules such as iCAM-1, selectins and CD11/CD18 [37, 38]. However, the exact role of neutrophils in renal I/R is not well-understood and results so far have been contradictory. Inhibition of neutrophil infiltration was found to be effective in reducing experimental renal injury after I/R [37]. However, this finding could not be confirmed in another *in vivo* study [39]. Monocytes migrate to injured tissues where they differentiate into either resident mature macrophages (M1) or dendritic cells. Macrophages invade mouse renal tissue within 1 hour after reperfusion, and persist for several days [40]. During the early phase of kidney I/R, large amounts of reactive oxygen and nitrogen intermediates and pro-inflammatory cytokines (e.g. IL-1β and TNF-α) are produced by M1 macrophages and drive a polarized Th1 immune response which contributes to injury. In mice, depletion of kidney and spleen macrophages prior to renal I/R injury prevented AKI, whereas adoptive transfer of macrophages reversed this effect [41]. In order to evaluate the effect of hAAT treatment on leukocyte influx, we analyzed the amount of macrophages and granulocytes in the post-ischemic kidneys of control and hAAT-treated mice. As previously described [42], granulocytes infiltrated the interstitial space within the first hours after 20-min ischemic period. Interstitial granulocytes peaked at 24h post-op, subsequently receding to baseline levels. On the contrary, macrophages gradually infiltrated the intertubular space over the course of the first 3 days after kidney reperfusion. Although the inhibitory effect of hAAT on neutrophil migration is well documented [13, 43], levels of early pro-inflammatory mediators and neutrophil influx were increased by hAAT soon after peritonitis in a recent *in vivo* study [44]. In our experimental conditions, hAAT did not have a significant effect on neutrophil recruitment in post-ischemic kidneys, most likely due to the inability of hAAT to inhibit renal iCAM expression (S3 Fig). In contrast, macrophage influx significantly decreased in hAAT-treated animals at day 1 post-op. A similar inhibitory effect of hAAT on macrophage infiltration and function has been described in different inflammatory models [13, 22].

The kidney has strong regenerative capacity after injury. In contrast to most other organs such as heart and brain, the post-ischemic kidney is able to completely restore its function and structure after a mild AKI. However, when the injury is more severe, AKI can lead to incomplete tubular regeneration, proliferation of fibroblasts, chronic tubulointerstitial inflammation and excessive build-up of extracellular matrix, with progression to chronic disease [45]. Therefore, the fibrotic process occurring after acute tubular injury can have severe clinical consequences [46, 47]. TGF-β superfamily members have been extensively linked to renal fibrosis [48] since they can control the transcription of genes related to fibrotic processes including those that encode collagens. MMP-9 is also known to be involved in various inflammatory and chronic kidney diseases [49], especially in obstructive nephropathy [50]. KIM-1 is another such promoter of kidney fibrosis, which can link acute renal damage to chronic kidney disease (CKD) [51]. KIM-1 levels are enhanced in CKD [52–54], and is expressed in fibrotic and inflamed tissue [55]. Interestingly, in human allografts KIM-1 expression correlates with interstitial fibrosis [56] and higher KIM-1 urine levels predict long-term renal graft loss [57]. Therefore, KIM-1 might play an important role in the development of progressive kidney disease. Although hAAT treatment did not have a significant effect either on renal collagen deposition or on TGF-β and MMP-9 gene expression, urine KIM-1 levels at day 15 after I/R injury were significantly decreased by this serine protease inhibitor. However, this reduction of KIM-1 was not reflected by a reduction of maladaptative kidney repair in our experimental model. The lack of difference in renal fibrosis indicates that the effect of hAAT on renal injury in our experimental conditions was modest, and short-lived due to antibody formation against hAAT in mice.

Similar to what has been observed in previous studies [13, 58], repeated i.p. injections of hAAT induced a strong humoral immune response against this human protein in C57Bl/6 mice. Mouse anti-hAAT antibodies were already detected at day 8 post-op. In mice, hAAT activity is limited *in vivo* as it leads to rapid anti-hAAT antibody production after hAAT administration [13]. The lack of effect of hAAT on adverse renal remodeling could be explained by the early anti-hAAT antibody formation in our experimental setting. Interestingly, we observed a positive correlation between anti-hAAT antibody formation and urine KIM-1 levels at day 15 after kidney reperfusion (Fig 5B). Positive effects of hAAT on adverse tissue remodeling have been described previously in a mouse model of ischemic myocardial injury using the same dose regime [9]. However, in that study CD1 mice were used and anti-hAAT antibody formation was not evaluated.

Our study provides evidence of the protective effect (albeit modest) of hAAT on renal I/R injury in mice and supports the potential of this natural protein to ameliorate ischemic and inflammatory conditions. We found a significant decrease in plasma NGAL and macrophage infiltration during the first 24 hours, and a significant decrease in plasma urea levels and tubular necrosis at 48 hours. This supports a protective effect of hAAT demonstrated on four biological levels (inflammatory cell infiltration, release of tubular cell damage marker, reduced glomerular filtration, and histological injury). These differences were statistically significant despite the small number of animals used and substantial variation. However, the observed effect size was limited and one could speculate that a longer ischemia time, causing more severe damage, would have given a more marked result. Therefore, the clinical relevance of our findings should be evaluated carefully. Given the amount of AAT required for our experiments, it was not feasible to extract such a quantity from mouse plasma nor to produce recombinant mouse AAT. In constrast, AAT purified from pooled human plasma is already commercially available as a therapeutic agent and approved at a dose of 60 mg/kg weekly by the Food and Drug Administration for replacement therapy [59]. The remarkably favorable 20-year safety profile of hAAT [60] has led to an expansion of its experimental use, highlighting the potential of this natural occurring protein as an anti-inflammatory and immunomodulatory agent. Recently, a recombinant form of AAT which appears to have stronger anti-inflammatory properties has become available [61]. Currently, clinical trials with hAAT are performed in acute graft-versus-host disease (GvHD), diabetes mellitus type I, and acute myocardial infarction. Preliminary results from trials in type 1 diabetes patients indicate that hAAT was safe and well-tolerated in pediatric subjects [62], and improved β-cell function reducing the need for insulin after hAAT treatment in recently diagnosed patients [63]. In addition, administration of Prolastin^®^ in patients with ST-segment elevation myocardial infarction was also well-tolerated and decreased the acute inflammatory response [64]. The short-term protection against I/R injury provided by hAAT in our experimental model could positively impact graft outcome after organ transplantation. New therapies to reduce I/R injury of allografts are especially welcome when they are devoid of side effects. hAAT is a natural agent with proven safety in the treatment of patients who are AAT deficient. Further studies are warranted to confirm the beneficial effects of hAAT therapy in humans which would support the design of a clinical trial with hAAT in settings of renal I/R injury such as kidney transplantation.

## Supporting information

S1 Fig Effect of hAAT (80 mg/kg/day; i.p.) treatment on renal gene expression after I/R Injury. Relative mRNA expression of IL-1β, MGL-1, MIP-1α, MCP-1, IRF5, CXCR2 and KC in post-ischemic kidneys at 2h and day 1 post-op. Relative gene expression expressed as Rq (fold change) = 2^-ΔΔCt^. Grey boxes represent the control group treated with hAlb. White boxes represent the group treated with hAAT. Mann-Whitney U test (two-tailed) (n = 6–8 animals per group).
(PDF)

S2 Fig Effect of hAAT (80 mg/kg/day; i.p.) treatment on kidney fibrosis after I/R Injury (IRI). **A.** Score of fibrotic area in post-ischemic kidneys at day 8 and 15 post-op. Quantification of collagen-stained area (μm^2^) was performed in non-overlapping fields (*x200* magnification) throughout 2 different regions of the kidney using the image software KS 400. Two-way analysis of variance (ANOVA) followed by *Bonferroni* post-test. **B-E.** Picro-Sirius Red-stained frontal sections of mouse kidney. **B, D** representative images of ischemic kidney of a control mouse at different time-points after IRI. **C, E** representative images of ischemic kidney of a mouse treated with hAAT at different time-points after IRI. OC = outer cortex; CMJ = cortico-medullary junction. Scale bar = 100 μm (original magnification *x100*). **F.** Relative mRNA expression of col-1α, col-4, MMP-9 and TGF-β in post-ischemic kidneys at day 8 and 15 post-op. Relative gene expression expressed as Rq (fold change) = 2^-ΔΔCt^. Grey boxes represent the control group treated with hAlb. White boxes represent the group treated with hAAT. Mann-Whitney U test (two-tailed) (*n* = 6–8 animals per group).
(PDF)

S3 Fig Effect of hAAT (80 mg/kg/day; i.p.) treatment on protein iCAM-1 expression in post-ischemic kidneys during the early phase of I/R Injury. Kidney sections were stained with a CD54 monoclonal anti-iCAM-1 antibody (eBioscience, dilution 1:75). **A.** Representative image of ischemic control mouse kidney. **B.** Representative image of ischemic mouse kidney treated with hAAT. Scale bar = 50 μm (original magnification *x200*; dark green = autofluorescence of the tubuli; bright green = iCAM-1 protein expression).
(PDF)

S1 Table Sequence of primers. (PDF)

## References

[1] Chung KY, Park JJ, Kim YS. The role of high-mobility group box-1 in renal ischemia and reperfusion injury and the effect of ethyl pyruvate. Transplant Proc. 2008;40(7): 2136–8. 10.1016/j.transproceed.2008.06.040 18790172

[2] Carell RW. The molecular structure and pathology of alpha 1-antitrypsin. Lung. 1990;168 Suppl: 530–4. 2117161 10.1007/BF02718175

[3] Massi G, Chiarelli C. Alpha 1-antitrypsin: molecular structure and the Pi system. Acta Paediatr. 1994;393: 1–4. 10.1111/j.1651-2227.1994.tb13197.x 8032110

[4] Travis J, Salvesen GS. Human plasma proteinase inhibitors. Annu Rev Biochem. 1983;52: 655–709. 10.1146/annurev.bi.52.070183.003255 6193754

[5] Wenger RH, Rolfs A, Marti HH, Bauer C, Gassmann M. Hypoxia, a novel inducer of acute phase gene expression in a human hepatoma cell line. J Biol Chem. 1995;270(46): 27865–70. 7499259 10.1074/jbc.270.46.27865

[6] Brunetti ND, Correale M, Pellegrino PL, Cuculo A, Biase MD. Acute phase proteins in patients with acute coronary syndrome: Correlations with diagnosis, clinical features, and angiographic findings. Eur J Intern Med. 2007;18(2): 109–17. 10.1016/j.ejim.2006.07.031 17338962

[7] Gilutz H, Siegel Y, Paran E, Cristal N, Quastel MR. Alpha 1-antitrypsin in acute myocardial infarction. Br Heart J. 1983;49(1): 26–9. 6600394 10.1136/hrt.49.1.26 PMC485205

[8] Zager RA, Johnson AC, Frostad KB. Rapid renal alpha-1 antitrypsin gene induction in experimental and clinical acute kidney injury. PLoS One. 2014;9(5): e98380 10.1371/journal.pone.0098380 24848503 PMC4029978

[9] Toldo S, Seropian IM, Mezzaroma E, Van Tassell BW, Salloum FN, Lewis EC, et al Alpha-1 antitrypsin inhibits caspase-1 and protects from acute myocardial ischemia-reperfusion injury. J Mol Cell Cardiol. 2011;51(2): 244–51. 10.1016/j.yjmcc.2011.05.003 21600901

[10] Lewis EC. Expanding the clinical indications for alpha(1)-antitrypsin therapy. Mol Med. 2012;18: 957–70. 10.2119/molmed.2011.00196 22634722 PMC3459478

[11] Subramaniyam D, Virtala R, Pawlowski K, Clausen IG, Warkentin S, Stevens T, et al TNF-alpha-induced self expression in human lung endothelial cells is inhibited by native and oxidized alpha1-antitrypsin. Int J Biochem Cell Biol. 2008;40(2): 258–71. 10.1016/j.biocel.2007.07.016 17869162

[12] Roelofs JJ, Rouschop KM, Leemans JC, Claessen N, de Boer AM, Frederiks WM, et al Tissue-type plasminogen activator modulates inflammatory responses and renal function in ischemia reperfusion injury. J Am Soc Nephrol. 2006;17(1): 131–40. 10.1681/ASN.2005010089 16291841

[13] Lewis EC, Shapiro L, Bowers OJ, Dinarello CA. Alpha1-antitrypsin monotherapy prolongs islet allograft survival in mice. Proc Natl Acad Sci U S A. 2005;102(34): 12153–8. 10.1073/pnas.0505579102 16093309 PMC1189344

[14] Rops AL, Figdor CG, van der Schaaf A, Tamboer WP, Bakker MA, Berden JH, et al The tetraspanin CD37 protects against glomerular IgA deposition and renal pathology. Am J Pathol. 2010;176(5): 2188–97. 10.2353/ajpath.2010.090770 20348240 PMC2861084

[15] Raats CJ, Bakker MA, Hoch W, Tamboer WP, Groffen AJ, van den Heuvel LP, et al Differential expression of agrin in renal basement membranes as revealed by domain-specific antibodies. J Biol Chem. 1998;273(28): 17832–8. 9651386 10.1074/jbc.273.28.17832

[16] Nijenhuis T, Sloan AJ, Hoenderop JG, Flesche J, van Goor H, Kistler AD, et al Angiotensin II contributes to podocyte injury by increasing TRPC6 expression via an NFAT-mediated positive feedback signaling pathway. Am J Pathol. 2011;179(4): 1719–32. 10.1016/j.ajpath.2011.06.033 21839714 PMC3181349

[17] Perico N, Cattaneo D, Sayegh MH, Remuzzi G. Delayed graft function in kidney transplantation. Lancet. 2004;364(9447): 1814–27. 10.1016/S0140-6736(04)17406-0 15541456

[18] Alkhunaizi AM, Schrier RW. Management of acute renal failure: new perspectives. Am J Kidney Dis. 1996;28(3): 315–28. 8804228 10.1016/s0272-6386(96)90487-4

[19] Dishart MK, Kellum JA. An evaluation of pharmacological strategies for the prevention and treatment of acute renal failure. Drugs. 2000;59(1): 79–91. 10718100 10.2165/00003495-200059010-00005

[20] Kunzendorf U, Haase M, Rolver L, Haase-Fielitz A. Novel aspects of pharmacological therapies for acute renal failure. Drugs. 2010;70(9): 1099–114. 10.2165/11535890-000000000-00000 20518578

[21] Ye J, Liao YT, Jian YQ, Zhang XD, Wei P, Qi H, et al Alpha-1-antitrypsin for the improvement of autoimmunity and allograft rejection in beta cell transplantation. Immunol Lett. 2013;150(1–2): 61–8. 10.1016/j.imlet.2013.01.009 23333354

[22] Shahaf G, Moser H, Ozeri E, Mizrahi M, Abecassis A, Lewis EC. alpha-1-antitrypsin gene delivery reduces inflammation, increases T-regulatory cell population size and prevents islet allograft rejection. Mol Med. 2011;17(9–10): 1000–11. 10.2119/molmed.2011.00145 21670848 PMC3188864

[23] Libert C, Van Molle W, Brouckaert P, Fiers W. alpha1-Antitrypsin inhibits the lethal response to TNF in mice. J Immunol. 1996;157(11): 5126–9. 8943423

[24] Lewis EC, Mizrahi M, Toledano M, DeFelice N, Wright JL, Churg A, et al Alpha1-antitrypsin monotherapy induces immunotolerance during islet allograft transplantation in mice. Proc Natl Acad Sci U S A. 2008;105(42): 16236–41. 10.1073/pnas.0807627105 18852465 PMC2566995

[25] Vaidya VS, Ramirez V, Ichimura T, Bobadilla NA, Bonventre JV. Urinary kidney injury molecule-1: a sensitive quantitative biomarker for early detection of kidney tubular injury. Am J Physiol Renal Physiol. 2006;290(2): F517–29. 10.1152/ajprenal.00291.2005 16174863

[26] Mishra J, Dent C, Tarabishi R, Mitsnefes MM, Ma Q, Kelly C, et al Neutrophil gelatinase-associated lipocalin (NGAL) as a biomarker for acute renal injury after cardiac surgery. Lancet. 2005;365(9466): 1231–8. 10.1016/S0140-6736(05)74811-X 15811456

[27] Mishra J, Ma Q, Prada A, Mitsnefes M, Zahedi K, Yang J, et al Identification of neutrophil gelatinase-associated lipocalin as a novel early urinary biomarker for ischemic renal injury. J Am Soc Nephrol. 2003;14(10): 2534–43. 14514731 10.1097/01.asn.0000088027.54400.c6

[28] Han WK, Bailly V, Abichandani R, Thadhani R, Bonventre JV. Kidney Injury Molecule-1 (KIM-1): a novel biomarker for human renal proximal tubule injury. Kidney Int. 2002;62(1): 237–44. 10.1046/j.1523-1755.2002.00433.x 12081583

[29] Heyne N, Kemmner S, Schneider C, Nadalin S, Konigsrainer A, Haring HU. Urinary neutrophil gelatinase-associated lipocalin accurately detects acute allograft rejection among other causes of acute kidney injury in renal allograft recipients. Transplantation. 2012;93(12): 1252–7. 10.1097/TP.0b013e31824fd892 22513480

[30] Kohei J, Ishida H, Tanabe K, Tsuchiya K, Nitta K. Neutrophil gelatinase-associated lipocalin is a sensitive biomarker for the early diagnosis of acute rejection after living-donor kidney transplantation. Int Urol Nephrol. 2013;45(4): 1159–67. 10.1007/s11255-012-0321-y 23161375

[31] Holzscheiter L, Beck C, Rutz S, Manuilova E, Domke I, Guder WG, et al NGAL, L-FABP, and KIM-1 in comparison to established markers of renal dysfunction. Clin Chem Lab Med. 2014;52(4): 537–546. 10.1515/cclm-2013-0693 24243749

[32] Eisner C, Faulhaber-Walter R, Wang Y, Leelahavanichkul A, Yuen PS, Mizel D, et al (2010). Major contribution of tubular secretion to creatinine clearance in mice. Kidney Int 77(6): 519–526. 10.1038/ki.2009.501 20032962 PMC3160625

[33] Waikar SS, Betensky RA, Bonventre JV (2009). Creatinine as the gold standard for kidney injury biomarker studies? Nephrol Dial Transplant 24(11): 3263–3265. 10.1093/ndt/gfp428 19736243

[34] Sharfuddin AA, Molitoris BA. Pathophysiology of ischemic acute kidney injury. Nat Rev Nephrol. 2011;7(4): 189–200. 10.1038/nrneph.2011.16 21364518

[35] Awad AS, Rouse M, Huang L, Vergis AL, Reutershan J, Cathro HP, et al Compartmentalization of neutrophils in the kidney and lung following acute ischemic kidney injury. Kidney Int. 2009;75(7): 689–98. 10.1038/ki.2008.648 19129795 PMC2656389

[36] Solez K, Morel-Maroger L, Sraer JD. The morphology of "acute tubular necrosis" in man: analysis of 57 renal biopsies and a comparison with the glycerol model. Medicine. 1979;58(5): 362–76. 481195

[37] Kelly KJ, Williams WW Jr., Colvin RB, Meehan SM, Springer TA, Gutierrez-Ramos JC, et al Intercellular adhesion molecule-1-deficient mice are protected against ischemic renal injury. J Clin Invest. 1996;97(4): 1056–63. 10.1172/JCI118498 8613529 PMC507153

[38] Rabb H, Mendiola CC, Dietz J, Saba SR, Issekutz TB, Abanilla F, et al Role of CD11a and CD11b in ischemic acute renal failure in rats. Am J Physiol. 1994;267(6 Pt 2): F1052–8. 7810691 10.1152/ajprenal.1994.267.6.F1052

[39] Thornton MA, Winn R, Alpers CE, Zager RA. An evaluation of the neutrophil as a mediator of in vivo renal ischemic-reperfusion injury. Am J Pathol. 1989;135: 509–515. 2782382 PMC1879883

[40] Li L, Huang L, Sung SS, Vergis AL, Rosin DL, Rose CE Jr., et al The chemokine receptors CCR2 and CX3CR1 mediate monocyte/macrophage trafficking in kidney ischemia-reperfusion injury. Kidney Int. 2008;74(12): 1526–37. 10.1038/ki.2008.500 18843253 PMC2652647

[41] Day YJ, Huang L, Ye H, Linden J, Okusa MD. Renal ischemia-reperfusion injury and adenosine 2A receptor-mediated tissue protection: role of macrophages. Am J Physiol Renal Physiol. 2005;288(4): F722–31. 10.1152/ajprenal.00378.2004 15561971

[42] Leemans JC, Stokman G, Claessen N, Rouschop KM, Teske GJ, Kirschning CJ, et al Renal-associated TLR2 mediates ischemia/reperfusion injury in the kidney. J Clin Invest. 2005;115(10): 2894–903. 10.1172/JCI22832 16167081 PMC1201659

[43] Gao W, Zhao J, Kim H, Xu S, Chen M, Bai X, et al alpha1-Antitrypsin inhibits ischemia reperfusion-induced lung injury by reducing inflammatory response and cell death. J Heart Lung Transplant. 2014;33(3): 309–15. 10.1016/j.healun.2013.10.031 24365768

[44] Kaner Z, Ochayon DE, Shahaf G, Baranovski BM, Bahar N, Mizrahi M, et al Acute Phase Protein alpha1-Antitrypsin Reduces the Bacterial Burden in Mice by Selective Modulation of Innate Cell Responses. J Infect Dis. 2014; 211(9): 1489–98. 10.1093/infdis/jiu620 25389308

[45] Yang L, Humphreys BD, Bonventre JV. Pathophysiology of acute kidney injury to chronic kidney disease: maladaptive repair. Contrib Nephrol. 2011;174: 149–55. 10.1159/000329385 21921619

[46] Venkatachalam MA, Griffin KA, Lan R, Geng H, Saikumar P, Bidani AK. Acute kidney injury: a springboard for progression in chronic kidney disease. Am J Physiol Renal Physiol. 2010;298(5): F1078–94. 10.1152/ajprenal.00017.2010 20200097 PMC2867413

[47] Coca SG, Yusuf B, Shlipak MG, Garg AX, Parikh CR. Long-term risk of mortality and other adverse outcomes after acute kidney injury: a systematic review and meta-analysis. Am J Kidney Dis. 2009;53(6): 961–73. 10.1053/j.ajkd.2008.11.034 19346042 PMC2726041

[48] Border WA, Noble NA. Transforming growth factor beta in tissue fibrosis. N Engl J Med. 1994;331(19): 1286–92. 10.1056/NEJM199411103311907 7935686

[49] Lenz O, Elliot SJ, Stetler-Stevenson WG. Matrix metalloproteinases in renal development and disease. J Am Soc Nephrol. 2000;11(3): 574–81. 10703682 10.1681/ASN.V113574

[50] Yang J, Shultz RW, Mars WM, Wegner RE, Li Y, Dai C, et al Disruption of tissue-type plasminogen activator gene in mice reduces renal interstitial fibrosis in obstructive nephropathy. J Clin Invest. 2002;110(10): 1525–38. 10.1172/JCI16219 12438450 PMC151817

[51] Humphreys BD, Xu F, Sabbisetti V, Grgic I, Naini SM, Wang N, et al Chronic epithelial kidney injury molecule-1 expression causes murine kidney fibrosis. J Clin Invest. 2013;123(9): 4023–35. 10.1172/JCI45361 23979159 PMC3755983

[52] Kuehn EW, Park KM, Somlo S, Bonventre JV. Kidney injury molecule-1 expression in murine polycystic kidney disease. Am J Physiol Renal Physiol. 2002;283(6): F1326–36. 10.1152/ajprenal.00166.2002 12388382

[53] van Timmeren MM, Bakker SJ, Vaidya VS, Bailly V, Schuurs TA, Damman J, et al Tubular kidney injury molecule-1 in protein-overload nephropathy. Am J Physiol Renal Physiol. 2006;291(2): F456–64. 10.1152/ajprenal.00403.2005 16467126

[54] Waanders F, van Timmeren MM, Stegeman CA, Bakker SJ, van Goor H. Kidney injury molecule-1 in renal disease. J Pathol. 2010;220(1): 7–16. 10.1002/path.2642 19921716

[55] van Timmeren MM, van den Heuvel MC, Bailly V, Bakker SJ, van Goor H, Stegeman CA. Tubular kidney injury molecule-1 (KIM-1) in human renal disease. J Pathol. 2007;212(2): 209–17. 10.1002/path.2175 17471468

[56] Schroppel B, Kruger B, Walsh L, Yeung M, Harris S, Garrison K, et al Tubular expression of KIM-1 does not predict delayed function after transplantation. J Am Soc Nephrol. 2010;21(3): 536–42. 10.1681/ASN.2009040390 20019169 PMC2831861

[57] van Timmeren MM, Vaidya VS, van Ree RM, Oterdoom LH, de Vries AP, Gans RO, et al High urinary excretion of kidney injury molecule-1 is an independent predictor of graft loss in renal transplant recipients. Transplantation. 2007;84(12): 1625–30. 10.1097/01.tp.0000295982.78039.ef 18165774 PMC2745062

[58] Grimstein C, Choi YK, Wasserfall CH, Satoh M, Atkinson MA, Brantly ML, et al Alpha-1 antitrypsin protein and gene therapies decrease autoimmunity and delay arthritis development in mouse model. J Transl Med. 2011;9: 21 10.1186/1479-5876-9-21 21345239 PMC3050720

[59] Gotzsche PC, Johansen HK. Intravenous alpha-1 antitrypsin augmentation therapy: systematic review. Dan Med Bull. 2010;57(9): A4175 20816015

[60] Petrache I, Hajjar J, Campos M. Safety and efficacy of alpha-1-antitrypsin augmentation therapy in the treatment of patients with alpha-1-antitrypsin deficiency. Biologics. 2009;3: 193–204. 19707408 10.2147/btt.2009.3088 PMC2726081

[61] Jonigk D, Al-Omari M, Maegel L, Muller M, Izykowski N, Hong J, et al Anti-inflammatory and immunomodulatory properties of alpha1-antitrypsin without inhibition of elastase. Proc Natl Acad Sci U S A. 2013;110(37): 15007–12. 10.1073/pnas.1309648110 23975926 PMC3773761

[62] Rachmiel M, Strauss P, Dror N, Benzaquen H, Horesh O, Tov N, et al Alpha-1 antitrypsin therapy is safe and well tolerated in children and adolescents with recent onset type 1 diabetes mellitus. Pediatr Diabetes. 2015. 10.1111/pedi.12283 26073583

[63] Gottlieb PA, Alkanani AK, Michels AW, Lewis EC, Shapiro L, Dinarello CA, et al alpha1-Antitrypsin therapy downregulates toll-like receptor-induced IL-1beta responses in monocytes and myeloid dendritic cells and may improve islet function in recently diagnosed patients with type 1 diabetes. J Clin Endocrinol Metab. 2014;99(8): E1418–26. 10.1210/jc.2013-3864 24527714 PMC4121034

[64] Abbate A, Van Tassell BW, Christopher S, Abouzaki NA, Sonnino C, Oddi C, et al Effects of Prolastin C (Plasma-Derived Alpha-1 Antitrypsin) on the acute inflammatory response in patients with ST-segment elevation myocardial infarction (from the VCU-alpha 1-RT pilot study). Am J Cardiol. 2015;115(1): 8–12. 10.1016/j.amjcard.2014.09.043 25456867

