# Supplementary material for: Human Alpha-1-Antitrypsin (hAAT) therapy reduces renal dysfunction and acute tubular necrosis in a murine model of bilateral kidney ischemia-reperfusion injury
Source: PLoS One. 2017 Feb 24;12(2):e0168981. doi: 10.1371/journal.pone.0168981 (PMC5325207; doi:10.1371/journal.pone.0168981)
Supplement: S1 Fig — Relative mRNA expression of IL-1β, MGL-1, MIP-1α, MCP-1, IRF5, CXCR2 and KC in post-ischemic kidneys at 2h and day 1 post-op. Relative gene expression expressed as Rq (fold change) = 2-ΔΔCt. Grey boxes represent the control group treated with hAlb. White boxes represent the group treated with hAAT. Mann-Whitney U test (two-tailed) (n = 6–8 animals per group). (PDF) [file pone.0168981.s001.pdf]

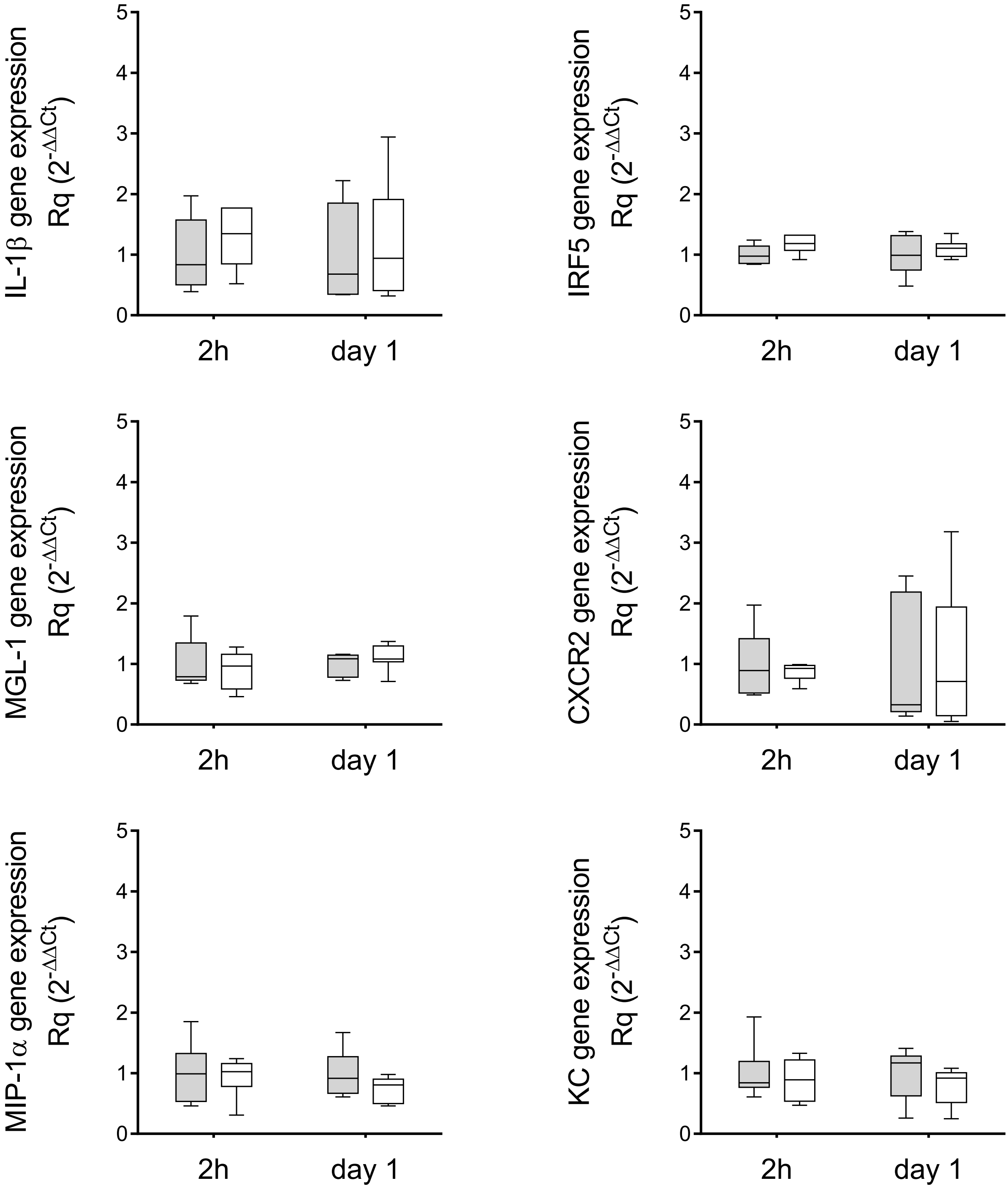

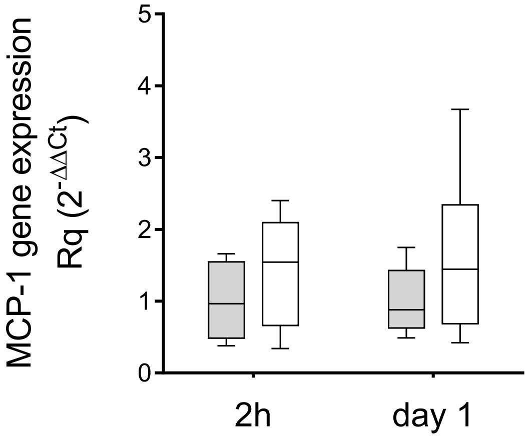

S1 Fig. Effect of hAAT (80 mg/kg/day; i.p.) treatment on renal gene expression after I/R injury. Relative mRNA expression of IL-1β, MGL-1, MIP-1α, MCP-1, IRF5, CXCR2 and KC in post-ischemic kidneys at 2h and day 1 post-op. Relative gene expression expressed as Rq (fold change) = $2^{-\Delta\Delta Ct}$. ■ = control group treated with hAlb. □ = group treated with hAAT. Mann-Whitney U test (two-tailed) (*n*=6-8 animals per group).
