# Supplementary material for: Human Alpha-1-Antitrypsin (hAAT) therapy reduces renal dysfunction and acute tubular necrosis in a murine model of bilateral kidney ischemia-reperfusion injury
Source: PLoS One. 2017 Feb 24;12(2):e0168981. doi: 10.1371/journal.pone.0168981 (PMC5325207; doi:10.1371/journal.pone.0168981)
Supplement: S2 Fig — A. Score of fibrotic area in post-ischemic kidneys at day 8 and 15 post-op. Quantification of collagen-stained area (μm2) was performed in non-overlapping fields (x200 magnification) throughout 2 different regions of the kidney using the image software KS 400. Two-way analysis of variance (ANOVA) followed by Bonferroni post-test. B-E. Picro-Sirius Red-stained frontal sections of mouse kidney. B, D representative images of ischemic kidney of a control mouse at different time-points after IRI. C, E representative images of ischemic kidney of a mouse treated with hAAT at different time-points after IRI. OC = outer cortex; CMJ = cortico-medullary junction. Scale bar = 100 μm (original magnification x100). F. Relative mRNA expression of col-1α, col-4, MMP-9 and TGF-β in post-ischemic kidneys at day 8 and 15 post-op. Relative gene expression expressed as Rq (fold change) = 2-ΔΔCt. Grey boxes represent the control group treated with hAlb. White boxes represent the group treated with hAAT. Mann-Whitney U test (two-tailed) (n = 6–8 animals per group). (PDF) [file pone.0168981.s002.pdf]

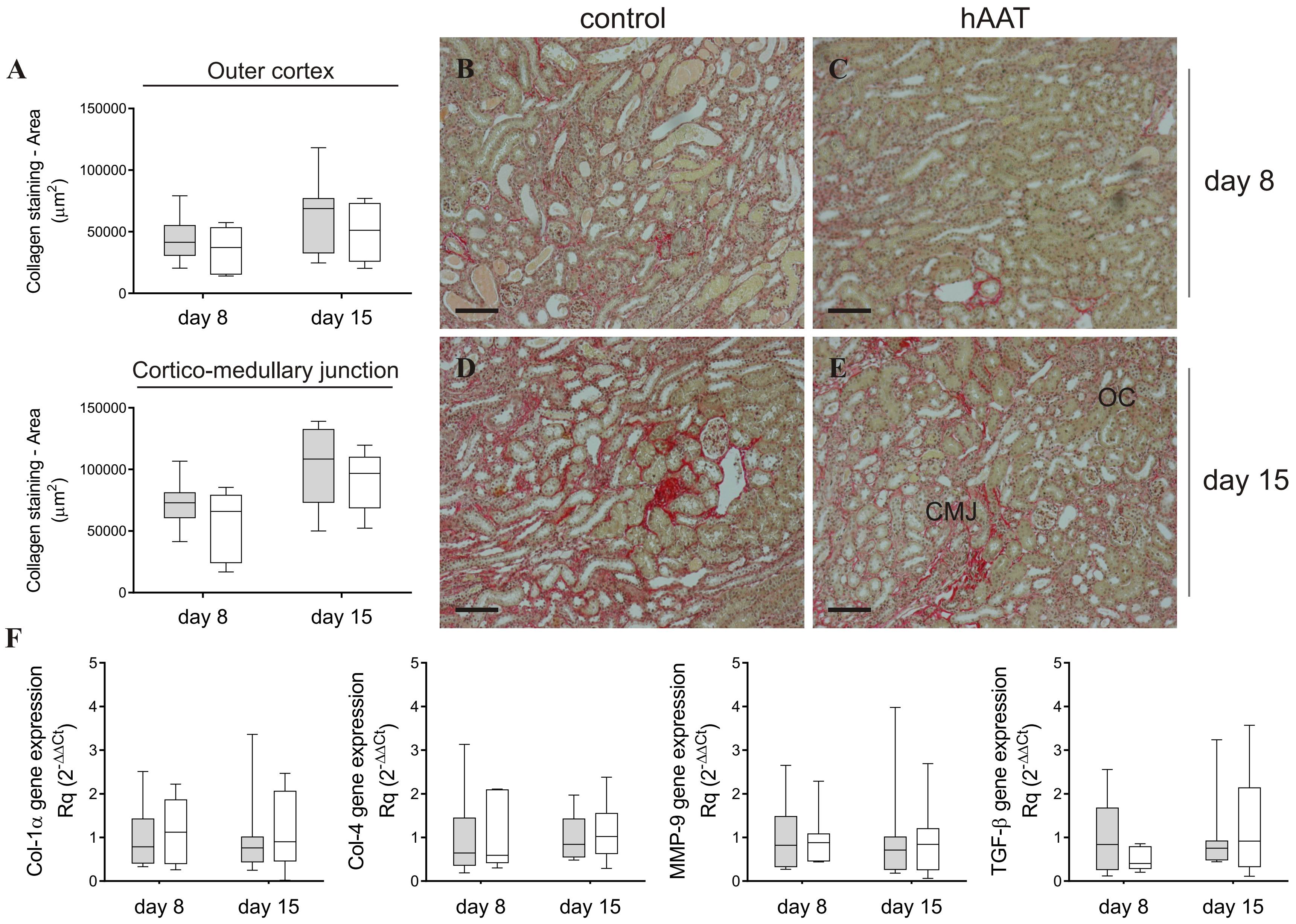

S2 Fig. Effect of hAAT (80 mg/kg/day; i.p.) treatment on kidney fibrosis after I/R injury (IRI). A. Score of fibrotic area in post-ischemic kidneys at day 8 and 15 post-op. Quantification of collagen-stained area (µm^2) was performed in non-overlapping fields (*x200* magnification) throughout 2 different regions of the kidney using the image software KS 400. Two-way analysis of variance (ANOVA) followed by *Bonferroni* post-test. **B-E.** Picro-Sirius Red-stained frontal sections of mouse kidney. **B, D** representative images of ischemic kidney of a control mouse at different time-points after IRI. **C, E** representative images of ischemic kidney of a mouse treated with hAAT at different time-points after IRI. OC=outer cortex; CMJ=cortico-medullary junction. Scale bar = 100 µm (original magnification *x100*). **F.** Relative mRNA expression of col-1α, col-4, MMP-9 and TGF-β in post-ischemic kidneys at day 8 and 15 post-op. Relative gene expression expressed as Rq (fold change) = $2^{-\Delta\Delta Ct}$. ▒ = control group treated with hAlb. ☐ = group treated with hAAT. Mann-Whitney U test (two-tailed) (*n*=6-8 animals per group).
