# Supplementary material for: Human Alpha-1-Antitrypsin (hAAT) therapy reduces renal dysfunction and acute tubular necrosis in a murine model of bilateral kidney ischemia-reperfusion injury
Source: PLoS One. 2017 Feb 24;12(2):e0168981. doi: 10.1371/journal.pone.0168981 (PMC5325207; doi:10.1371/journal.pone.0168981)
Supplement: S3 Fig — Kidney sections were stained with a CD54 monoclonal anti-iCAM-1 antibody (eBioscience, dilution 1:75). A. Representative image of ischemic control mouse kidney. B. Representative image of ischemic mouse kidney treated with hAAT. Scale bar = 50 μm (original magnification x200; dark green = autofluorescence of the tubuli; bright green = iCAM-1 protein expression). (PDF) [file pone.0168981.s003.pdf]

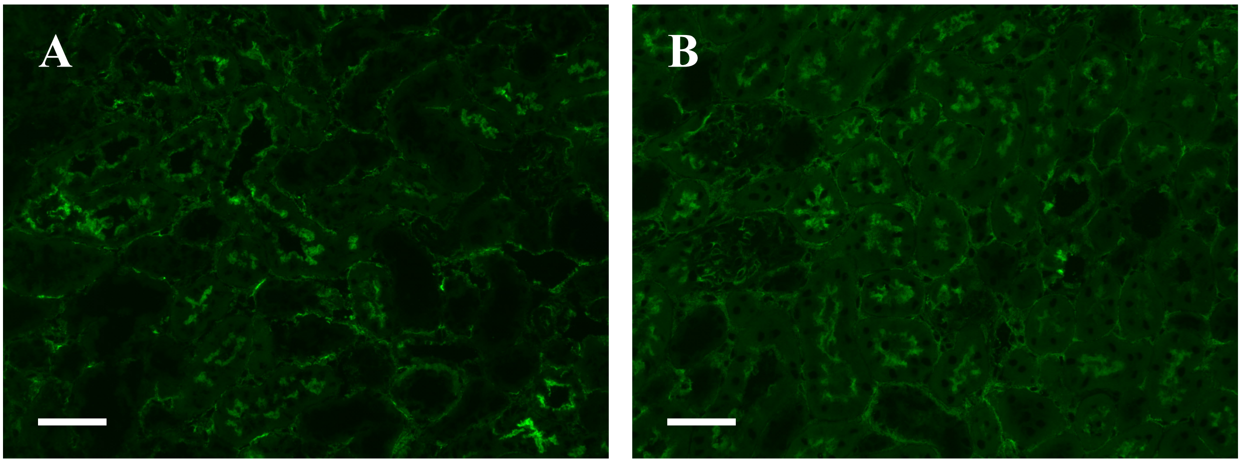

S3 Fig. Effect of hAAT (80 mg/kg/day; i.p.) treatment on protein iCAM-1 expression in post-ischemic kidneys during the early phase of I/R injury. Kidney sections were stained with a CD54 monoclonal anti-iCAM-1 antibody (eBioscience, dilution 1:75). **A.** Representative image of ischemic control mouse kidney. **B.** Representative image of ischemic mouse kidney treated with hAAT. Scale bar = 50 µm (original magnification *x200*; dark green = autofluorescence of the tubuli; bright green = iCAM-1 protein expression).
