# Supplementary material for: Human Alpha-1-Antitrypsin (hAAT) therapy reduces renal dysfunction and acute tubular necrosis in a murine model of bilateral kidney ischemia-reperfusion injury
Source: PLoS One. 2017 Feb 24;12(2):e0168981. doi: 10.1371/journal.pone.0168981 (PMC5325207; doi:10.1371/journal.pone.0168981)
Supplement: S1 Table — (PDF) [file pone.0168981.s004.pdf]

S1 Table. Sequence of primers.

Target gene	Primer sequence (5'-3')
IL-1β	AACGTGTGGGGGATGAATTG
	CATACTCATCAAAGCAATGT
MGL-1	TGAGAAAGGCTTTAAGAACTGGG
	GACCACCTGTAGTGATGTGGG
MIP-1α	TGAATGCCTGAGAGTCTTGG
	TTGGCAGCAAACAGCTTATC
MCP-1	GGATCAGAGATACTCATGAT
	GAGAAGATTACCTGAGTACA
IRF5	AAGGCCTGGGCTAAAGAGAC
	GCTTTTGTTAAGGGCACAGC
CXCR2	CCCAGAGTTTAGAACCCCCTATA
	TCTCCTCCACCTCTTCCTTTTAC
KC	ATAATGCCCTTTYACATTCTTTAACC
	AGTCCTTTGAACGTCTCTGTCC
TGF-β	GAGCGACGTCACTGGAGTTGTACG
	GATCCCGTTGATTTCCACGTGGAG
col-1α	CTTCACCTACAGCACCCTTGTG
	GAGTTTGGGTTGTTCGTCTGTTT
col-4	CAAATGCTTACAGCTTTTGGC
	TCTTCTCATGCACACTTGGC
MMP-9	CCTGGAACTCACACGACATCTTC
	CGGGTCAACTTCACATTCAAA
GAPDH	AGAAACCTGCCAAGTATGATGAC
	GTATTCATTGTCATACCAGGAAATGA
